# The Outcome of Thoracentesis versus Chest Tube Placement for Hepatic Hydrothorax in Patients with Cirrhosis: A Nationwide Analysis of the National Inpatient Sample

**DOI:** 10.1155/2017/5872068

**Published:** 2017-11-28

**Authors:** Ali Ridha, Yasir Al-Abboodi, Matthew Fasullo

**Affiliations:** ^1^University of Arkansas for Medical Science, 4301 West Markham Street, Little Rock, AR 72205, USA; ^2^Saint Davis Round Rock Medical Centre, 2400 Round Rock Ave, Round Rock, TX 78681, USA; ^3^Umass Memorial Medical Center, 55 Lake Avenue North, Worcester, MA 01655, USA

## Abstract

There are only a few studies with a small sample size of patients that have compared the risks of using chest tubes versus thoracentesis in hepatic hydrothorax. It has been shown that many complications may arise secondary to chest tube placement and is associated with increased morbidity and mortality. In this retrospective study, patients with cirrhosis were identified from the 2009 National Inpatient Sample by using ICD-9 codes; we evaluated the risk of chest tube versus thoracentesis in a largest population with hepatic hydrothorax to date to measure the mortality and the length of stay. A total of 140,573 patients with liver cirrhosis were identified. Of this, 1981 patients had a hepatic hydrothorax and ended up with either thoracentesis (1776) or chest tube (205). The mortality in those who received a chest tube was two times higher than that in thoracentesis group with a *P* value of ≤0.001 (CI 1.43–312). In addition, the length of hospital stay of the chest tube group was longer than that of the thoracentesis subset (7.2 days versus 3.8 days, resp.). We concluded that chest tube placement has two times higher mortality rate and longer hospital length of stay when compared to patients who underwent thoracentesis.

## 1. Introduction

Cirrhosis is the end stage of chronic liver disease, which occurs due to gradual hepatic fibrosis leading to the irreversible destruction of liver anatomy. Liver cirrhosis has a high morbidity and mortality, which is the 14th most common cause of death all over the world and the 4th in central Europe. It leads to more than 1 million deaths per year in the world, and greater than 150,000 deaths per year in Europe [1]. The most common causes of liver cirrhosis are alcohol, chronic viral infection (hepatitis B or C), and nonalcoholic steatohepatitis (NASH) [2]. In early stages, the condition may be reversible by treating the underlying cause, but in later stages, liver transplant will be the ultimate therapy [3].

Shortness of breath and hypoxia are very common in patients with liver cirrhosis for a multitude of reasons [4]. It can be due to the mechanical impingement of the ascetic fluid on the diaphragm, hepatopulmonary syndrome [5], portopulmonary hypertension [6], and cirrhotic cardiomyopathy [7].

Hepatic hydrothorax is one of the complications of liver cirrhosis, which may cause shortness of breath as well, occurring in less than 10% of cirrhotic patients with known portal hypertension [8]. The ascetic fluid will spread into the chest at the pleural cavity via a defect in the diaphragm and usually accumulating in the pleural space of the right lung [9].

Patients are usually advised to limit the salt and fluid intake along with diuretic use [10, 11]. Some patient may need thoracentesis [12], transjugular intrahepatic portosystemic shunt placement [13], pleurodesis [14], thoracoscopic surgery to repair diaphragmatic defects [15], or liver transplantation [16]. The following article will discuss and review the risk of the complications of chest tubes compared with thoracentesis in cirrhotic patients with hepatic hydrothorax.

## 2. Method

The 2009 National Inpatient Sample (NIS) was used to create a panel of patients diagnosed with cirrhosis. The NIS is one of the largest publicly available database that has more than 7 million admissions from nationally distributed all over the united states including private, academic, nonfederal, and public hospitals. The NIS is part of the Healthcare Cost and Utilization Project (HCUP) by the Agency for Healthcare Research and Quality (AHRQ). Data including age, sex, race, length of stay, type of payers, admission types, primary and secondary diagnosis, hospital cost, hospital types and location, and other variables are included in the NIS.

### 2.1. Case and Outcome Variable Identifications

The ICD-9 (the clinical modification of the International Classification of Diseases) diagnostic codes were used to identify patients with history of liver cirrhosis (ICD-9 = 5718). The following ICD-9 codes were also used: chest tube placement (3404), thoracentesis (3491), hydrothorax (5118), and transudate pleural effusion (5119). Other variables used in this study have already been identified in the NIS data, for instance, race and sex.

### 2.2. Statistical Analysis

The primary outcome of the study was examining the safety and the mortality difference of chest tube placement versus thoracentesis in people with cirrhosis complicated by hepatic hydrothorax. We also assessed the mortality difference of chest tube placement versus thoracentesis in cirrhotic versus noncirrhotic patients. Data was reviewed and analyzed using the IBM SPSS version 24 statistical software for Mac computer. The target population analyzed was cirrhotic patients with hydrothorax or transudate pleural effusion-related admissions. The target population was further subdivided into people who had chest tube placement for drainage versus those who had thoracentesis. Mean, percentages, and standard deviation of the mean were used to examine the demographic feature of the target population. A multivariate logistic regression statistical test was used to compare the mortality and morbidity difference between these two groups. A confidence interval (CI) of 95% and *P* value less than 0.05 were determined to define the statistical difference between the two groups.

## 3. Result

A total of 140,573 patients with diagnosis of cirrhosis were identified using ICD-9 code. Only 1981 patients out of 140,573 had transudate pleural effusion or hydrothorax and underwent a thoracentesis or chest tube during their hospitalization. 1776 had a thoracentesis (diagnosis and/or therapeutic) and 205 had a chest tube. 1121 (56.6%) were male and 860 (43.4%) were female (Table 1). 1240 (69.6%) were white, 154 (8.6%) black, 291 (16.3%) Hispanic, 48 (2.7%) Asian, 10 (0.6%) Native American, and 39 (2.2%) others (Table 2). Mean length of stay was 7.2 ± 15.5 for a patient with chest tube and 3.8 ± 10 for a patient with thoracentesis. Mean age was 60.38 ± 15 versus 60 ± 13.4 in people with chest tube versus thoracentesis, respectively (Table 3). The inpatient mortality was two times greater in the chest tube group than in the thoracentesis group (odds ratio = 2.1; *P* value ≤ 0.001, CI 1.43–312). In addition, the length of stay of the chest tube group was longer than that of the thoracentesis group (7.2 days versus 3.8 days, resp.) (Table 3). The mortality was also 2 times higher in cirrhotic patients who had chest tube comparing to noncirrhotic patients who had chest tube (odds ratio = 2, *P* value ≤ 0.001, CI 1.39–2.9). The mortality was 1.3 times higher in cirrhotic patients who had thoracentesis compared to noncirrhotic patients with thoracentesis (odds ratio 1.3, *P* value ≤ 0.01, CI 1.14–159). Comparing male versus female, cirrhotic patients who had a chest tube and or/thoracentesis had no significant mortality difference (value = 0.731, odds ratio = 0.97, and CI 0.71–1.21). Comparing white to black cirrhotic patients who had a chest tube and/or thoracentesis also had no significant mortality difference (*P* value = 0.127, odds ratio = 1.6, CI 0.8–3.1) (Table 4).

## 4. Discussion

Hepatic hydrothorax is a rare complication of liver cirrhosis. Less than 10% of patients with severe liver disease may end up with hepatic hydrothorax, which commonly presents as shortness of breath, and low oxygen saturation and can be complicated by underlying infection. The development of a hydrothorax is most likely due to the spread of the ascetic fluid, accumulating from portal hypertension, into the pleural space due to small diaphragmatic defects [17]. Following emergence of symptoms, usually a simple chest X-ray is usually sufficient to point towards the diagnosis. Ultimately, a thoracentesis will be needed for a fluid sample to demonstrate a transudate fluid, like the peritoneal fluid with a greater protein concentration as pleura has a higher capacity for reabsorption for water than in the peritoneum [18].

Rarely, hydrothorax patients may end up with an acute tension hydrothorax, which generally leads to an acute change in respiratory status along with hypotension [19]. Another complication of hepatic hydrothorax is spontaneous bacterial empyema, which can happen around two percent of cirrhotic patients and can be seen in up to 16% when patients develop a hepatic hydrothorax [20].

Once a hydrothorax is diagnosed, the first line of therapy includes salt restriction, fluid restriction, and diuresis [21]. Generally, guidelines following failure of these options are slightly more ambiguous. When shortness of breath progresses to hypoxia due to the accumulation of hydrothorax, a thoracentesis is a fast and useful tool for relieving the symptom [22]. A patient who continues to decompensate following the interventions should be assessed for liver transplantation to reduce the risks of complications. Treatment options for recurrent hepatic hydrothorax that are not candidates for transplant include TIPS, repeated thoracentesis, and pleurodesis [23].

Multiple small-power studies have discussed the complications of chest tube placement for hepatic hydrothorax such as subcutaneous emphysema, organ puncture, haemothorax due to artery laceration, pulmonary edema from rapid removal of fluid (reexpansion pulmonary edema), misplacement of chest tube, protein and electrolyte depletion, infection, bleeding, and death [8, 24–26].

In 2016, Sharaf-Eldin et al. devised a new technique by using a pigtail catheter with a small caliber (22 gauge) for treating recurrent hepatic hydrothorax and, also, concluded that chest tube insertion for hepatic hydrothorax is associated with high complication rate and should be avoided [26].

One major flaw with these prior studies is the small sample used for most them. Our study contains the largest sample size analyzed for complications and mortality of chest tubes in patients with hepatic hydrothorax. We demonstrate a clear increase in mortality as well as hospital length of stay in patients who received a chest tube compared to those who are managed with a thoracentesis (diagnostic or therapeutic). We hope that by presenting this data, we can reiterate the increased morbidity and mortality using chest tubes for hepatic hydrothorax. We advocate for the use of repeated thoracentesis, TIPS, and, if feasible, liver transplant as the only therapeutic options for this condition.

Our study had several limitations that are inherent in the use of an electronic administrative database (NIS), which lacks the details available in standardized clinical trials. Long-term outcomes, complications, indications, severity of cirrhosis (MELD or Child-Pugh Score), and rehospitalization rates could not be assessed using the NIS database. The outcome of the procedure, degree of procedural difficulties, and provider experience could not be assessed.

## 5. Conclusion

Chest tube placement for hepatic hydrothorax has two times higher mortality and longer length of stay compared to those who underwent thoracentesis.

## Figures and Tables

**Table 1:** Gender.

		Chest tube	Thoracentesis	Total
ISEX				
Male	Count	113	1008	1121
	%	55.1%	56.8%	56.6%
Female	Count	92	768	860
	%	44.9%	43.2%	43.4%
Total	Count	205	1776	1981
	%	100.0%	100.0%	100.0%

**Table 2:** Race.

		Chest tube	Thoracentesis	Total
Race (uniform)				
White	Count	131	1109	1240
	%	72.4%	69.3%	69.6%
Black	Count	15	139	154
	%	8.3%	8.7%	8.6%
Hispanic	Count	30	261	291
	%	16.6%	16.3%	16.3%
Asian	Count	2	46	48
	%	1.1%	2.9%	2.7%
Native American	Count	1	9	10
	%	0.6%	0.6%	0.6%
Others	Count	2	37	39
	%	1.1%	2.3%	2.2%
Total	Count	181	1601	1782
	% within	100.0%	100.0%	100.0%

**Table 3:** Length of stay.

	Mean length of stay (days) S.E.		Mean	Age	S.E.
Chest tube in cirrhotic patient	7.21	15.56	60.38		15.43
Thoracentesis in cirrhotic patient	3.8	10.44	60.61		13.48

**Table 4:** Mortality difference and binary logistic regression.

Mortality difference	S.E.	*P* value	Odds ratio	CI of 95%
Chest tube versus thoracentesis in patients with cirrhosis	0.119	≤0.001	2.11	1.43–3.12
Male versus female cirrhotic patients who have chest tube and/or thoracentesis	0.14	0.731	0.97	0.71–1.21
White versus black cirrhotic patients who have chest tube and/or thoracentesis	0.32	0.127	1.6	0.8–3.1
